# Efficacy and safety of efgartigimod as an add-on therapy in patients with NMOSD and MOGAD at the acute attack phase

**DOI:** 10.3389/fimmu.2026.1793153

**Published:** 2026-05-12

**Authors:** Weifan Yin, Shige Wang, Wei Lu, Tianjiao Duan

**Affiliations:** 1 Department of Neurology, The Second Xiangya Hospital, Central South University, Changsha, Hunan, China; 2 Clinical Medical Research Center for Stroke Prevention and Treatment of Hunan Province, Department of Neurology, The Second Xiangya Hospital, Central South University, Changsha, Hunan, China; 3 Faculty of Biology, Medicine and Health, University of Manchester, Manchester, United Kingdom

**Keywords:** acute attack, add-on therapy, efgartigimod, myelin oligodendrocyte glycoprotein associated disease, neuromyelitis optica spectrum disorders

## Abstract

**Background:**

Neuromyelitis optica spectrum disorders (NMOSD) and myelin oligodendrocyte glycoprotein-associated disease (MOGAD) are autoimmune antibody-mediated diseases. Efgartigimod is a neonatal Fc receptor-targeting therapeutic that causes a reduction in the antibody titers. The efficacy and the safety of efgartigimod as an add-on therapy with intravenous methylprednisolone (IVMP) in patients with NMOSD and MOGAD were assessed in this study.

**Methods:**

In total, 27 adult patients diagnosed with NMOSD or MOGAD were enrolled, with 13 patients treated with IVMP plus efgartigimod and 14 comparable controls treated with IVMP alone. Efgartigimod was administered intravenously at 10 mg/kg at four doses or 20 mg/kg at two doses. The Expanded Disability Status Scale (EDSS) scores, the serum immunoglobulin G (IgG) levels, and the pathogenic antibody titers were evaluated before and after therapy.

**Results:**

Compared with IVMP alone, the efgartigimod group achieved better outcome with EDSS reduction of 1.3 ± 0.6 (*p* < 0.05) compared with the control group (0.5 ± 0.5). In the efgartigimod-treated group, the serum IgG levels decreased by 69.8% after therapy (*p* < 0.001), and nine patients (69.2%) showed a reduction in antibody titers. Moreover, the EDSS and antibody titers showed a rapid downward trend in the intensive therapy cohort (20 mg/kg, two doses).

**Conclusions:**

In this preliminary study, efgartigimod add-on therapy showed better trends than IVMP alone in accelerating short-term recovery in patients with NMOSD and MOGAD at the acute phase.

## Introduction

Neuromyelitis optica spectrum disorders (NMOSD) and myelin oligodendrocyte glycoprotein-associated disease (MOGAD) are acknowledged autoimmune-mediated demyelinating disorders involving the central nervous system (CNS). The prominent characteristic of these diseases is their recurrent nature, leading to severe paralysis and blindness (1–4). Aquaporin-4 (AQP4), immunoglobulin G (IgG), and myelin oligodendrocyte glycoprotein (MOG) IgG were thought to be pathogenic, resulting in clinical attacks and relapses (5, 6).

Although the prognosis of MOGAD is better than that of NMOSD, the core symptoms caused by acute optic neuritis and acute myelitis are irreversible and disabling. Thus, timely and aggressive treatment during acute attacks is crucial in order to alleviate CNS damage and reduce neurologic deficits. Intravenous methylprednisolone (IVMP) is generally considered as the first-line therapy for acute relapses in both NMOSD and MOGAD (7, 8). However, in various retrospective studies, IVMP showed efficacy in only 50%–90% of patients with NMOSD and MOGAD (9, 10). Although the response to IVMP in MOGAD is better than that in NMOSD, recurrent episodes and inadequate treatment can still lead to poor recovery and even severe disability. Given this, add-on therapy concurrent with IVMP is recommended. Plasma exchange (PLEX) is used either as a rescue treatment in patients with glucocorticoid intolerance or combined with IVMP to achieve better efficacy (7, 8). Although PLEX has shown potential in removing antibodies, complements, and cytokines from the blood (11), there are still several limitations in clinical practice, including the plasma source, the invasive operation, the equipment requirement, and the high risk of allergic reactions. Furthermore, in a recent retrospective study that enrolled 22 patients with MOGAD and 20 patients with NMOSD, the use of IVMP in combination with PLEX failed to demonstrate better improvement than IVMP alone (10). Besides these regimens, immunoadsorption (IA) is an alternative method for the selective removal of certain IgG from blood, despite a lack of evidence from large-scale randomized controlled studies (12–14).

Previous studies have shown that IgG can be rescued from degradation in lysosomes by binding to the neonatal Fc receptor (FcRn) (15). Efgartigimod is an FcRn-targeting therapy that can block the binding of FcRn to IgG, reducing IgG to be recycled, thus downregulating the IgG levels in the blood (16, 17). In phase 1 and 2 trials, efgartigimod remarkably decreased the concentrations of all IgG subtypes without reducing the levels of other immunoglobulins (IgM and IgA) or of albumin (16, 18), indicating a more accurate and secure option than PLEX. Efgartigimod has been approved for the treatment of generalized myasthenia gravis (gMG) and chronic inflammatory demyelinating polyradiculoneuropathy (CIDP) (19, 20). Currently, it is globally applied as a potential treatment for IgG-mediated diseases, including Guillain–Barré syndrome (GBS) and autoimmune encephalitis (21–23). A number of cases and small cohorts also tentatively used efgartigimod as an add-on therapy in patients with NMOSD at the acute phase; however, clinical data are still scarce. MOGAD cases are particularly unreported at present (24–27). Therefore, more real-world studies are needed to evaluate the efficacy and safety of efgartigimod in NMOSD and MOGAD during acute attacks.

## Methods

### Patient enrollment

This retrospective study was conducted in the Second Xiangya Hospital of Central South University. Patients were collected from December 2023 to August 2024. A total of 42 adult patients met the diagnostic criteria for NMOSD or MOGAD and underwent an acute attack. For this study, the main inclusion criteria were: i) meeting the diagnostic criteria for NMOSD (International Panel for NMO Diagnosis) in 2015 or MOGAD (International MOGAD Panel proposed criteria) in 2023; ii) an acute attack defined as inflammatory lesions of the optic nerve or spinal cord resulting in the corresponding neurological dysfunction; iii) seropositivity for AQP4-IgG or MOG-IgG at this attack; and iv) 2.5 ≤ Expanded Disability Status Scale (EDSS) score ≤ 8 during this attack. The main exclusion criteria were: i) severe disease state with unsuitability for this study (e.g., requiring ventilatory support or coma state); ii) use of monoclonal antibodies or other biologicals within 6 months; and iii) administered intravenous immunoglobulin (IVIG) or PLEX within 4 weeks.

**Figure 1:**
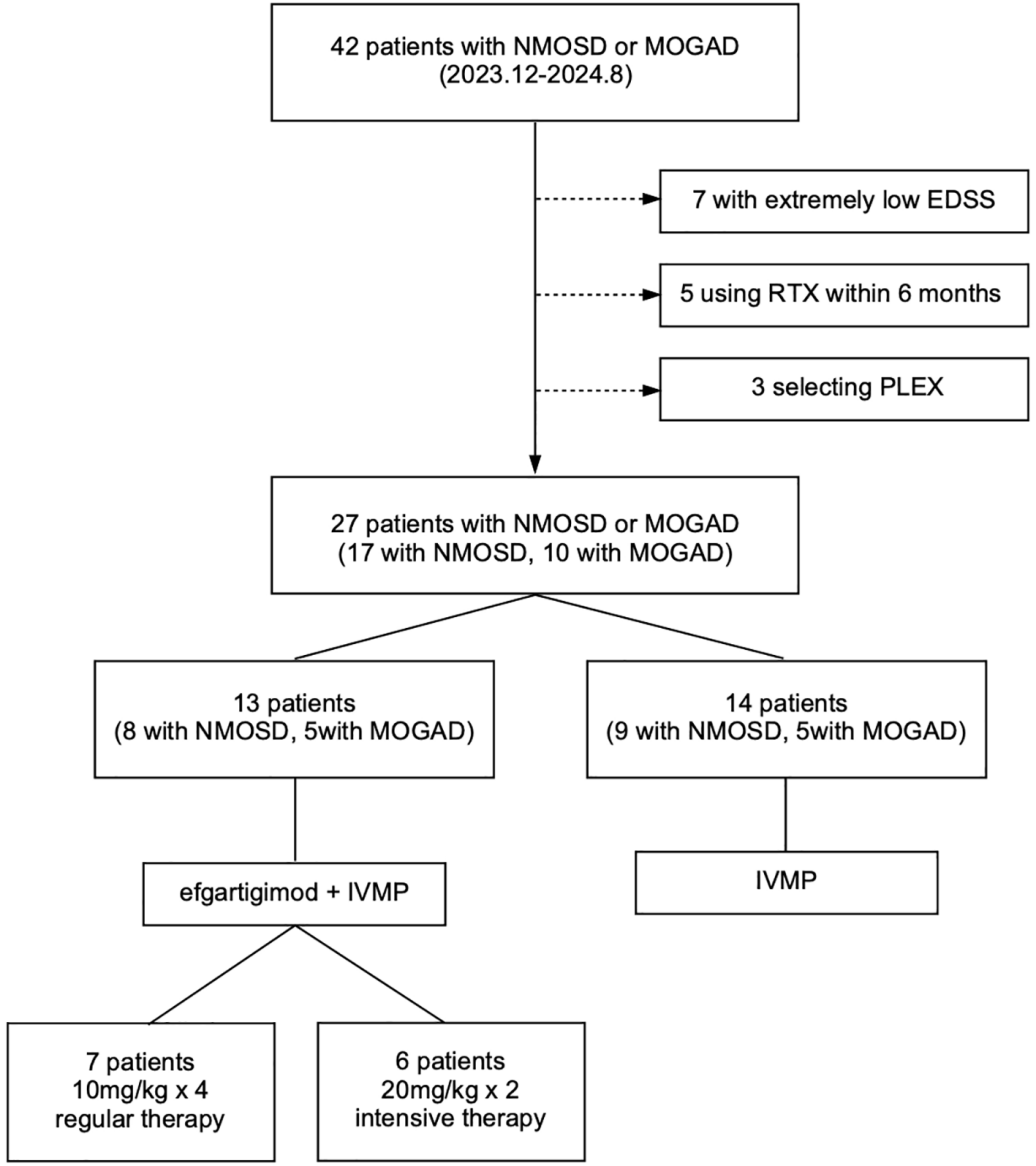
Patient enrollment process. *NMOSD*, neuromyelitis optica spectrum disorders; *MOGAD*, myelin oligodendrocyte glycoprotein-associated disease; *PLEX*, plasma exchange; *RTX*, rituximab; *EDSS*, Expanded Disability Status Scale.

After screening, 15 patients were excluded due to extremely low EDSS scores, selection of other therapies, or the use of biologicals within 6 months. Of the remaining 27 participants, 13 patients (eight with NMOSD and five with MOGAD) chose IVMP plus efgartigimod as treatment during the acute attack period, while 14 patients (nine with NMOSD and five with MOGAD) chose IVMP alone. The grouping was based entirely on the voluntariness of the patients to receive efgartigimod or not. IVMP treatment was standardized (1,000 mg for 3 days, 500 mg for 3 days, 240 mg for 3 days, and 120 mg for 3 days, then tapering gradually). The process of enrollment is presented in **Figure 1**. All clinical data were collected after signed informed consent. The study protocol was approved by the Ethics Committee of the Second Xiangya Hospital, Central South University. The clinical study followed the Declaration of Helsinki.

### Efgartigimod administration

Among the 13 patients who received efgartigimod, seven patients were randomly assigned to the regular therapy cohort, while the remaining six were in the intensive therapy cohort. Efgartigimod was administered intravenously once weekly at 10 mg/kg in four doses as regular therapy. Intensive therapy of efgartigimod was given intravenously on days 1 and 8 at a dose of 20 mg/kg.

### Data collection and patient evaluation

For the 27 enrolled patients, baseline parameters were collected, including age, sex, onset age, clinical subtype, serum antibody titers, and previous treatments. Therapeutic efficacy was evaluated weekly using the EDSS scores (blinded assessors) from pretreatment (baseline) to 4 weeks after the initial treatment. For the efgartigimod group, the serum IgG level was also assessed to evaluate effectiveness. Clinical safety was assessed before and after therapy based on patient responses, laboratory blood tests, and reported adverse events (AEs). Pathogenic antibody titers were measured at baseline and at 2 weeks after therapy to evaluate medication effects.

### Statistical analysis

Continuous variables are reported as the mean ± SD and were compared using an independent *t*-test or the Mann–Whitney *U* test. Categorical data are presented as percentage and were compared using *χ*^2^ or Fisher’s exact test. Statistical analyses were performed with SPSS version 25.0. Graphs were generated using GraphPad Prism version 9.5.1 software. Differences were considered statistically significant at a two-tailed *p* < 0.05.

## Results

### Baseline clinical characteristics

A total of 27 adult patients with NMOSD or MOGAD (13 in group 1 treated with efgartigimod and 14 in group 2 as controls) were enrolled in this study. The baseline clinical characteristics are shown in **Table 1**. The NMOSD-to-MOGAD ratios were 8:5 in group 1 and 9:5 in group 2. The mean patient age at this attack was 48.5 ± 11.8 years (range = 25–65 years) in group 1 and was 40.7 ± 13.7 years (range = 23–61 years) in group 2. The sex ratios were 92.3% (group 1) and 64.3% (group 2). The average numbers of episodes were 3.1 ± 1.4 (group 1) and 2.9 ± 1.4 (group 2). Subtypes of the clinical features included optic neuritis (ON) (76.9% in group 1 and 64.3% in group 2), transvers myelitis (TM) (53.8% in group 1 and 64.3% in group 2), and brainstem presentations (23.1% in group 1 and 14.3% in group 2).

**Table 1:** Baseline clinical characteristics of the antibody-mediated central nervous system demyelinating syndrome patients.

Clinical variable	Mean ± SD or *N* (%) or range		*P*-value
	IVMP+efgartigimod (*n* = 13)	IVMP alone (*n* = 14)	
Age (years)	48.5 ± 11.8	40.7 ± 13.7	0.126
Sex (female, %)	12 (92.3%)	9 (64.3%)	0.165
Frequency of attacks (*n*)	3.1 ± 1.4	2.9 ± 1.4	0.788
Clinical classification			
Optic neuritis	10 (76.9%)	9 (64.3%)	0.678
Transverse myelitis	7 (53.8%)	9 (64.3%)	0.704
Brainstem presentations	3 (23.1%)	2 (14.3%)	0.648
Previous treatment			
Prednisone	12 (92.3%)	11 (78.6%)	0.596
Azathioprine	5 (38.5%)	7 (50.0%)	0.704
Mycophenolate mofetil	5 (38.5%)	5 (35.7%)	1.000
Cyclophosphamide	1 (7.7%)	0	0.481
Rituximab	4 (30.8%)	4 (28.6%)	1.000
Ripertamab	1 (7.7%)	0	0.481
Initiation status			
Baseline EDSS scores at relapse	4.9 ± 1.3	4.6 ± 2.1	0.651
Antibody titers (range) at relapse	1:32 to 1:3,200	1:32 to 1:1,000	0.298

*IVMP*, intravenous methylprednisolone; *EDSS*, Expanded Disability Status Scale.

In previous treatments, oral prednisone was administered in 92.3% of the patients in group 1 and 78.6% of those in group 2. All patients received traditional immunosuppressants (IS) or biologics therapy. Before efgartigimod initiation, the baseline EDSS scores were 4.9 ± 1.3 (group 1) and 4.6 ± 2.1(group 2), and the pathologic antibody titers ranged from 1:32 to 1:3,200 (group 1) and from 1:32 to 1:1,000 (group 2). Thus, the disease classification and severity were comparable between the two groups.

### Clinical effect to efgartigimod

**Figure 2:**
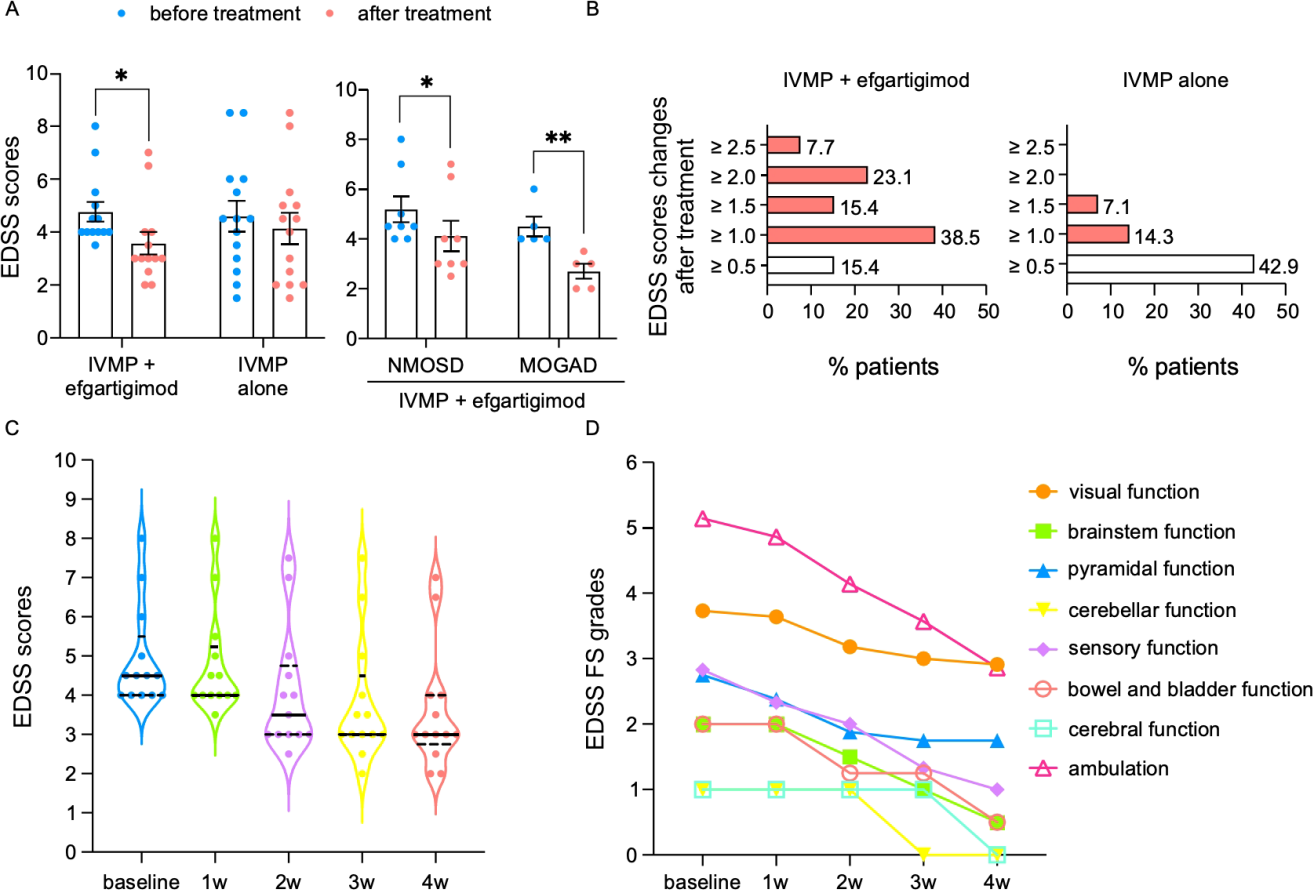
**(A)** Changes in the Expanded Disability Status Scale (EDSS) scores in the intravenous methylprednisolone (IVMP) plus efgartigimod group and the IVMP alone group before and after treatment, with EDSS improving in the neuromyelitis optica spectrum disorders (NMOSD) and myelin oligodendrocyte glycoprotein-associated disease (MOGAD) subgroups before and after treatment. **(B)** Improvement in the EDSS scores after treatment presented as percentage of patients. **(C, D)**. Changes in the EDSS scores **(C)** and individual functional system grades **(D)** in efgartigimod-treated NMOSD and MOGAD patients (*p < 0.05 and **p < 0.01).

During the acute attack, IVMP was applied as the standard regimen. Efgartigimod was used as an add-on therapy to alleviate symptoms and to reduce the serological antibody titers. The changes in the EDSS scores in the two groups before and after treatment are shown in **Figure 2A**. When the treatment was completed, the EDSS scores achieved were 3.6 ± 1.6 in the IVMP plus efgartigimod group (*p* < 0.05) and 4.1 ± 2.2 in the IVMP alone group. In the efgartigimod group, the EDSS scores in patients with MOGAD (*n* = 5) decreased from 4.5 ± 0.87 to 2.7 ± 0.67 (*p* < 0.01), which showed better improvement than the NMOSD subgroup (from 5.19 ± 1.49 to 4.13 ± 1.71, *n* = 8, *p* < 0.05) (**Figure 2A**). On the other hand, 84.7% of the patients in the IVMP plus efgartigimod group and 21.4% in the IVMP alone group had an improvement by at least 1 point of the EDSS score after treatment (**Figure 2B**).

We further analyzed the changes in the EDSS scores and the individual functional systems (FS) weekly in the efgartigimod therapy group. Improvement in the EDSS scores started at 2 weeks after the administration of efgartigimod (4.1 ± 1.6) and peaked at 3 weeks from initiation (3.8 ± 1.6) (**Figure 2C**). In each FS grade, the walking ability was remarkably ameliorated by 2.3 points (*n* = 7), the sensory function by 1.5 points (*n* = 6), and the visual function by 0.8 points (*n* = 11) (**Figure 2D**). The cerebral and cerebellar function entirely returned to normal.

### Immune indicator response to treatment

In the efgartigimod treatment group (*n* = 13), the serum IgG levels were assessed weekly, while the serologic pathogenic antibody titers were measured at baseline and at 2 weeks after treatment completion.

**Figure 3:**
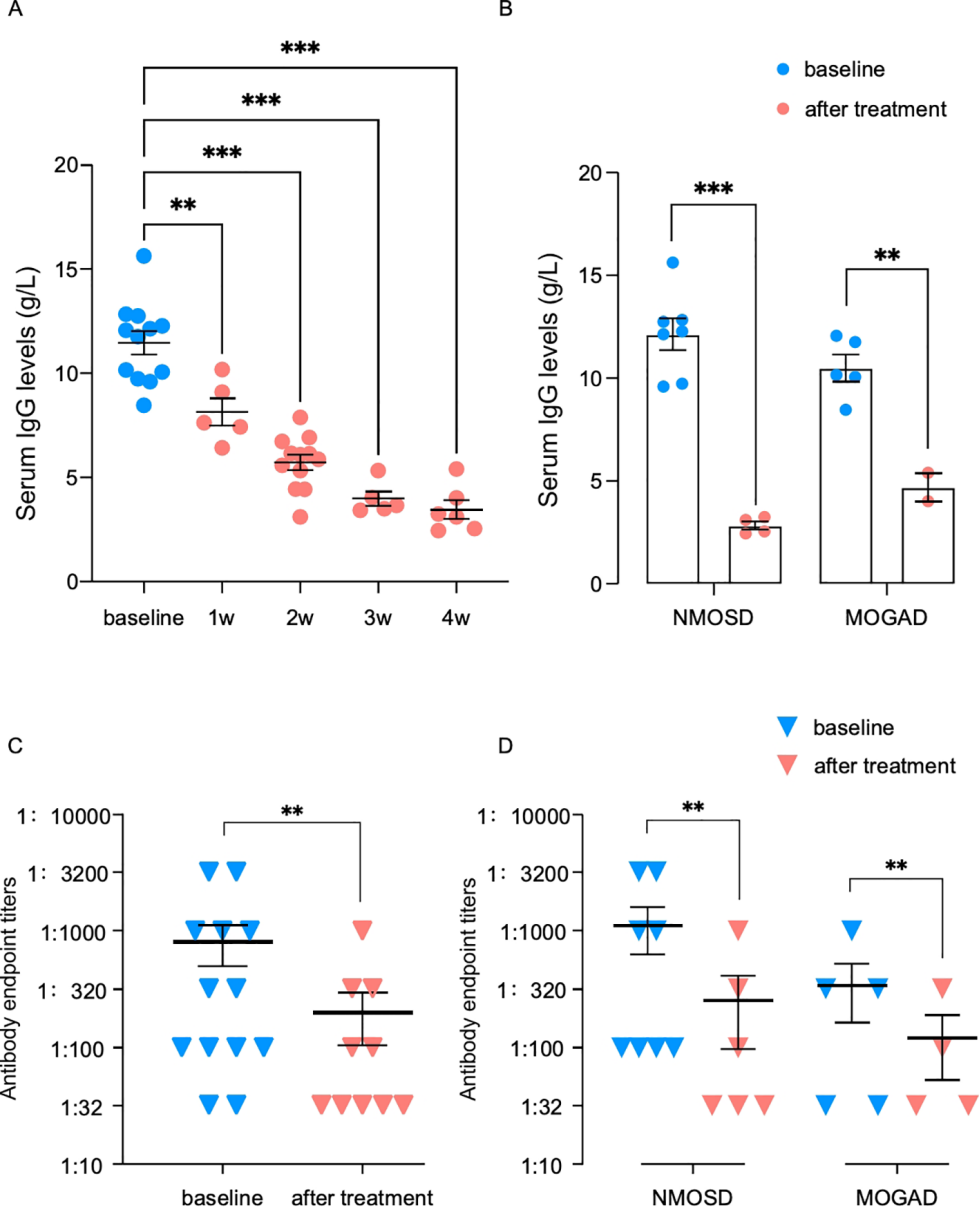
**(A)** Weekly changes in the serum immunoglobulin G (IgG) levels in efgartigimod-treated patients. **(B)** Levels of serum IgG at baseline and after treatment with efgartigimod in the neuromyelitis optica spectrum disorders (NMOSD) and myelin oligodendrocyte glycoprotein-associated disease (MOGAD) subgroups. **(C)** Overall changes in the serum pathogenic antibody titers in efgartigimod-treated patients. **(D)** Respective antibody titer changes at baseline and after treatment with efgartigimod in the NMOSD and MOGAD subgroups. Comparison of the levels at baseline and after treatment (**p < 0.01 and ***p < 0.001).

Judging from the downward trend of the whole group, the mean serum IgG level at baseline was 11.5 g/L, decreasing by 26.1% (8.2 g/L) at week 1 after the first dose (*p* < 0.01), which plateaued at week 3 and then reached a maximum reduction of 69.8% (3.5 g/L) at week 4 (*p* < 0.001) (**Figure 3A**). Assessment of the two subgroups separately showed that the serum IgG levels decreased from 12.1 to 2.8 in the NMOSD subgroup (*p* < 0.001) and from 10.5 to 4.7 in the MOGAD subgroup (*p* < 0.01) (**Figure 3B**).

At baseline, all patients were examined for AQP4-IgG or MOG-IgG titers. Among them, two patients showed notable high titers of 1:3,200, while the other two had relatively low titers of 1:32. The titers of the remaining patients ranged from 1:100 to 1:1,000. At 2 weeks after efgartigimod treatment completion, 10 patients were retested for serum antibodies. The antibody titers were substantially lower than the baseline levels (*p* < 0.01). There were five patients who reached quite low titers of 1:32, and only one case maintained a high titer of 1:1,000, with a previous titer of 1:3,200 (**Figure 3C**). In the NMOSD subgroup, the antibody titers decreased in all tested patients (*n* = 6, *p* < 0.01). However, in the MOGAD subgroup, one patient with a low titer (1:32) maintained a consistent level, with the other three patients tested for antibodies showing decreasing levels (*n* = 4, *p* < 0.01) (**Figure 3D**).

### Subcohort comparison and analysis

**Figure 4:**
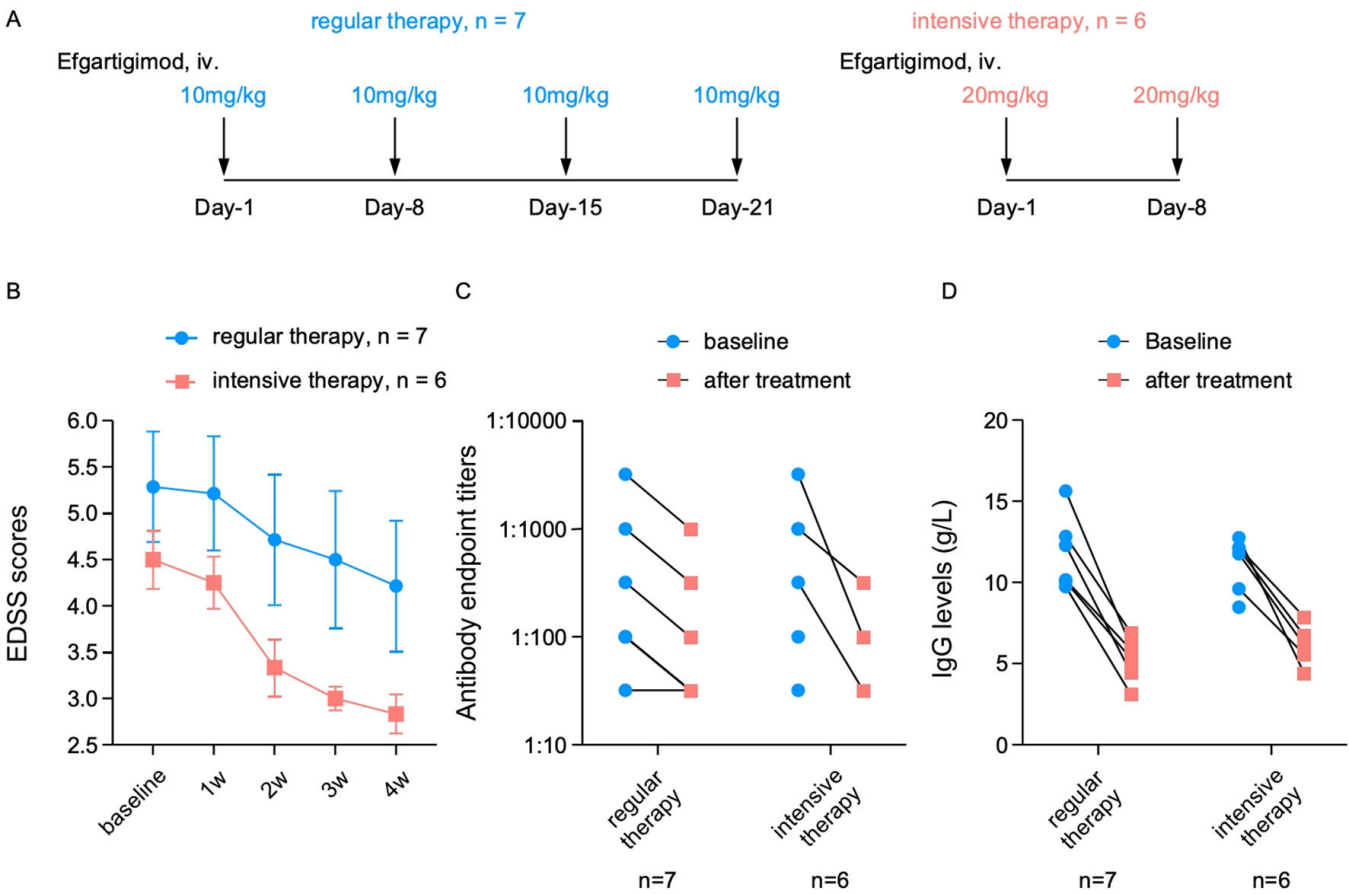
**(A)** Schematic diagram of the therapeutic dose and frequency of efgartigimod in the regular and intensive therapy cohorts. **(B–D)** Comparisons of the Expanded Disability Status Scale (EDSS) score reductions **(B)**, antibody titer changes **(C)**, and serum immunoglobulin G (IgG) level variations **(D)** in the regular and intensive therapy cohorts.

As planned, we randomly assigned an equal number of patients in the efgartigimod group (*n* = 13) into the regular and intensive therapy cohorts. The dosage and the medication intervals are illustrated in **Figure 4A**. The overall EDSS scores in the intensive therapy cohort decreased more rapidly and largely, from 4.5 at baseline to 3.3 at week 2, then to 2.8 at week 4. However, the baseline score of the regular therapy cohort was 5.3, which declined to 4.7 at week 2 and to 4.2 at week 4 (**Figure 4B**).

To estimate the differences in immune response, we compared the changes in the individual serum antibody titers and IgG levels between the two cohorts. The titers of six patients in the regular therapy cohort (*n* = 7) showed varying reductions, except for one case that showed constant titers (from 1:32 to 1:32). In these six patients, three had titers decreasing from 1:100 to 1:32, while the other three showed titer levels two times lower than the baseline. However, in the intensive therapy group (*n* = 6), follow-up antibody tests were performed only in three patients. The titers of these patients showed significant reductions, with two or three times lower values than the baseline (**Figure 4C**). The declining trends in the serum IgG levels were found to be similar between the two groups, from 11–12 g/L at baseline to 5–6 g/L after treatment (**Figure 4D**).

### Safety data of efgartigimod

No severe AEs were observed during the study. Mild to moderate AEs included transient headache or dizziness in two patients without treatment and mild hepatic damage in four patients resolved by polyunsaturated phosphatidylcholine.

**Figure 5:**
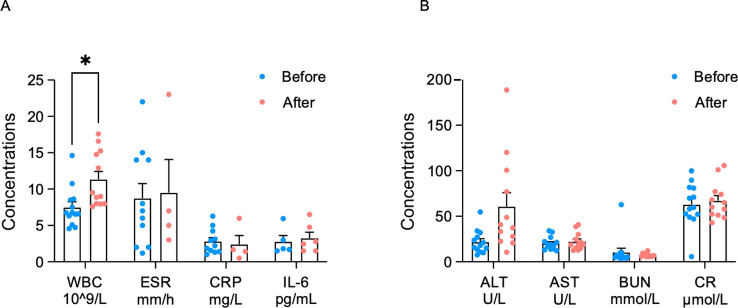
Changes in the inflammatory markers **(A)** and hepatorenal function **(B)** before and after efgartigimod treatment in patients with neuromyelitis optica spectrum disorders (NMOSD) and myelin oligodendrocyte glycoprotein-associated disease (MOGAD) (*p < 0.05). *WBC*, white blood cells; *ESR*, erythrocyte sedimentation rate; *CRP*, C-reactive protein; *IL-6*, interleukin-6; *ALT*, alanine transaminase; *AST*, aspartate aminotransferase; *BUN*, blood urea nitrogen; *CR*, creatinine.

Infections were the most frequently reported AEs during the treatment periods. In our study, five patients had leukocytosis without manifestations of infection (*p* < 0.05), which was associated with IVMP (**Figure 5A**). Other inflammatory markers including the erythrocyte sedimentation rate, C-reactive protein, and interleukin-6 showed no significant differences compared with the baseline levels.

The hepatorenal function was monitored by blood biochemicals. Alanine transaminase (ALT) increased to the upper limit of the normal range after treatment, but without statistical difference (**Figure 5B**). After the administration of hepatoprotective drugs, ALT returned to the normal range within 1–2 weeks. Aspartate aminotransferase, blood urea nitrogen, and creatinine were maintained at stable levels.

## Discussion

NMOSD and MOGAD are recognized IgG-mediated diseases. Several studies have supported that the early clearance of pathogenic IgG may act as an effective approach for these autoimmune diseases (7, 8). Efgartigimod, a human IgG1 antibody Fc fragment, has been engineered to increase affinity to endosomal FcRn than to endogenous IgG. Efgartigimod can prevent endogenous IgG binding to FcRn, resulting in IgG degradation and serum IgG level reduction (16, 17). The fast-acting effect of efgartigimod in gMG is well understood; however, its efficacy and safety in other IgG-mediated disorders, such as NMOSD and MOGAD, is still less studied. In this study, we analyzed the efficacy and safety of efgartigimod as an add-on strategy in patients with NMOSD and first provided these data in patients with MOGAD.

The enrolled patients (*n* = 27) were characterized by multiple attacks (approximately three times), received various IS (100% of the total), and suffered slight to moderate disability (average EDSS >4.5 at baseline). During acute attacks, add-on therapy with efgartigimod in these patients is highly necessary in order to reduce pathogenic antibodies and alleviate symptoms. In terms of efficacy, the EDSS scores in the efgartigimod group decreased by 1.3 on average at week 4 (*p* < 0.05), which was better than that of the control group (0.5 on average). Previous studies showed EDSS reductions of 0.365–0.5 after IVMP alone (9, 28), which is consistent with our results. In addition, 84.7% of the patients in the efgartigimod group achieved EDSS improvements ≥1.0 points compared with only 21.4% in the IVMP alone group, indicating a large proportion of patients benefiting from efgartigimod treatment. Analysis of clinical amelioration using the FS grade showed that movement ability was the most prominent aspect, which could improve life quality in disabled patients. In terms of safety, asymptomatic leukocytosis was considered as the additive effect of IVMP. A transient increase of the liver enzymes was also related to IVMP, as glucocorticoid is metabolized through the liver while efgartigimod is catabolized by the proteolytic enzyme, which minimally affects liver and kidney function (16, 29).

The total serum IgG levels decreased by an average of 69.8% after a cycle use of efgartigimod, showing its excellent IgG degradation ability. The clearance of pathogenic IgG (AQP4-IgG or MOG-IgG) was also rapid and prominent. Of the patients retested for antibodies, 90% showed a reduction in titers, and more than half reached a low titer of 1:32 after add-on therapy. The most effective case showed an IgG titer reduction from 1:3,200 to 1:100. In terms of antibody clearance, the percentage of efgartigimod exceeded the 85% of PLEX in antibody reduction (8). In the analyses of the NMOSD and MOGAD subgroups, the improvement in EDSS appears to be better in MOGAD (*p* < 0.01) than in NMOSD (*p* < 0.05). However, when evaluating the immune indicators, we found that the decreases in the ranges of IgG levels and the percentage of antibody titers were lower in the MOGAD subgroup. Therefore, the preferable clinical outcome of MOGAD than NMOSD was due to the nature of the disease having good prognosis rather than the antibody clearance therapy. Another small retrospective study of FcRn inhibitor showed good results in terms of EDSS and AQP4-IgG reduction when adopting IVMP in combination with batoclimab therapy in nine patients with NMOSD (30). Although batoclimab has not been approved for clinical use, these results, together with the efgartigimod data, indicate a promising prospect of FcRn-targeting therapy in NMOSD and MOGAD.

To date, two cases have reported on the tentative application of efgartigimod (24, 25), showing good outcomes. A retrospective study compared efgartigimod plus IVMP with IVMP alone in the acute phase of NMOSD, which showed efgartigimod appearing to yield better results, but with no statistical differences presented due to the small group size (*n* = 4) (26). In these studies, the usage regimen of efgartigimod (10 mg/kg IV, weekly, four doses) originated from phase II and III clinical trials of gMG (18, 19). However, a number of clinical trials also attempted intensive or shock scheme of efgartigimod (20 mg/kg IV, weekly, two doses) to treat critical gMG patients or autoimmune encephalitis (29). In a recent study, Liu et al. conducted an efgartigimod use of 20 mg/kg on days 1 and 5 for the treatment of NMOSD (*n* = 13) during acute attacks and compared its efficacy with that of IVIG (27). The EDSS improvement was significant in the efgartigimod group and comparable to that of IVIG therapy. However, the FcRn-antagonizing potency of efgartigimod exceeded that of IVIG. Although all of these results suggest a possible good effect of efgartigimod, its optimal treatment regimen in NMOSD and MOGAD is still under discussion; in particular, no use has been reported in MOGAD. In this study, we attempted to compare regular (10 mg/kg, four doses) and intensive (20 mg/kg, two doses) therapies of efgartigimod in the same center. The total quantity of efgartigimod was the same, but the dosing interval differed. By comparing the EDSS scores and immune indicators, we found that the symptoms improved more rapidly during the first 2 weeks in the intensive group, and the reduction extent of the antibody titers in this group appeared to be larger (three cases refused antibody tests after treatment); however, the minimum IgG levels in the two groups were similar at week 4. These results might advocate an intensive treatment of efgartigimod in the acute phase of disease. The faster clinical recovery may be attributed to the high-dose use of efgartigimod facilitating blood–brain barrier penetration and antibody clearance more effectively.

Humoral immunity mediated by autoimmune antibodies is involved in NMOSD and MOGAD development during acute attacks. Pathogenic antibody depletion was thought to be ideal for the treatment of these diseases. In our study, the use of efgartigimod at the acute phase helped to remarkably reduce the IgG levels and rapidly clear AQP4-IgG or MOG-IgG (2 weeks after treatment completion). We conducted a correlation analysis between IgG reduction (ΔIgG) and EDSS improvement (ΔEDSS). The results indicated a strong positive linear correlation between ΔIgG and ΔEDSS (*r* = 0.8192, *p* < 0.05) (*Supplementary data*). Thus, we believe that the decline in IgG is associated with clinical outcomes. The underlying mechanism might stem from the depletion of pathogenic IgG, which blocks the subsequent humoral cascade reactions, thus reducing pathological damage and improving clinical symptoms in a short period (4-week follow-up). In brief, the above mechanism and the fast-acting effect of efgartigimod make it a potential option for the management of NMOSD and MOGAD during acute attacks.

The limitations of this study should also be pointed out. Firstly, this is a retrospective cohort study with a small sample size, and the sample population was limited to a single center. Thus, selection and statistical bias could not be completely ruled out, and caution should be taken when generalizing the results to broader populations. The efficacy of efgartigimod as an add-on therapy needs further and large-scale studies to draw rigorous conclusions. Secondly, the data of the control group (IVMP alone) were insufficient. We did not ask the patients in this group for a retest of their antibody titers after treatment as, in our treatment experience, there would be no notable changes in the antibody titers in the short term with IVMP alone. Therefore, the partial comparison of the efficacy of immunotherapy between the two groups is insufficient. In addition, although the inclusion of patients with MOGAD and the comparison of two dosing regimens (regular and intensive cohort) bring innovation to this study, the cases in each subgroup were too small to conduct a reasonable statistical analysis, and we could only show some preliminary data to suggest clinical trends.

## Conclusion

This retrospective study offers clinically relevant and mechanistically coherent preliminary results showing the benefit and tolerability of add-on efgartigimod with IVMP in patients with NMOSD and MOGAD, which might accelerate short-term recovery during acute attacks. These results provide some new approaches and medication recommendations for clinicians. However, further evidence from randomized controlled trials is needed to confirm the efficacy and safety of the add-on therapy.

## Data Availability

The original contributions presented in the study are included in the article/**Supplementary Material**. Further inquiries can be directed to the corresponding author.
