# Supplementary figures and images for: Efficacy and safety of efgartigimod as an add-on therapy in patients with NMOSD and MOGAD at the acute attack phase
Source: Front Immunol. 2026 May 12;17:1793153. doi: 10.3389/fimmu.2026.1793153 (PMC13201218; doi:10.3389/fimmu.2026.1793153)

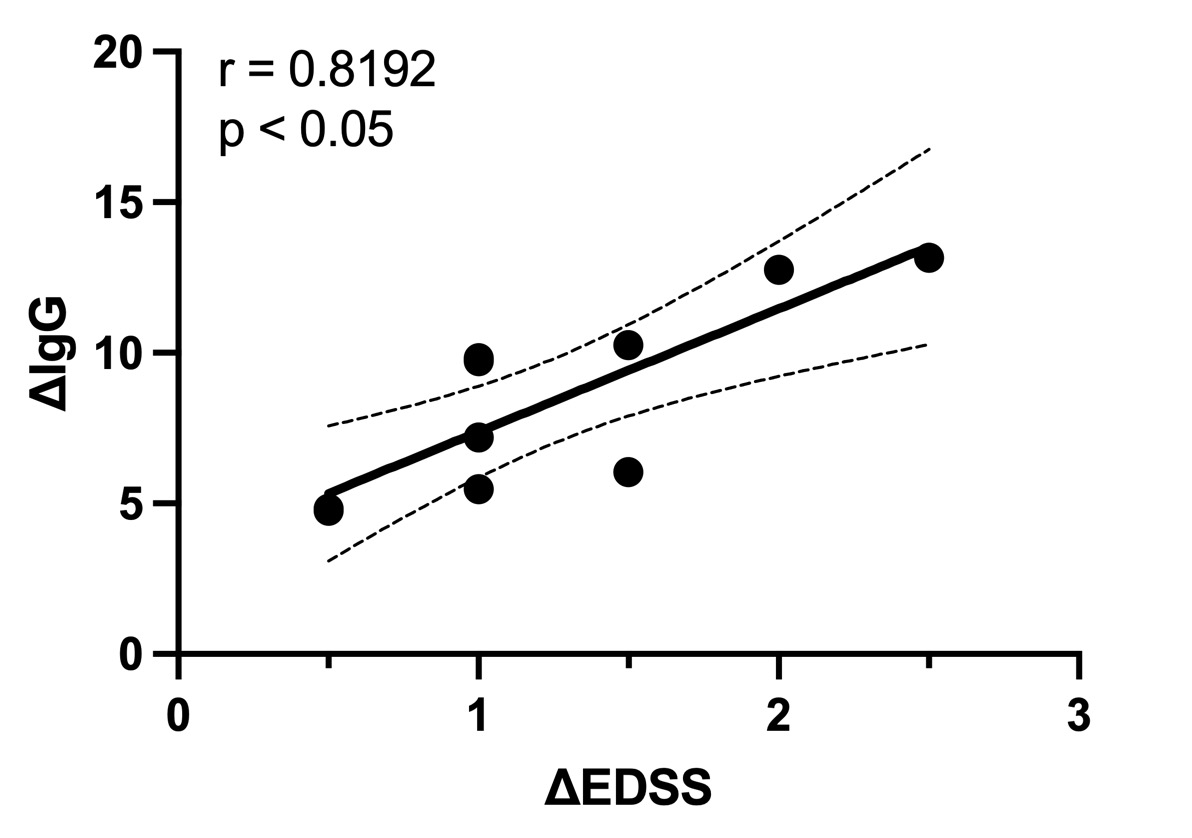

Supplement: Supplementary file 1 [file Image1.jpg]
